# Pathogenic mechanisms implicated in the intravascular coagulation in the lungs of BVDV-infected calves challenged with BHV-1

**DOI:** 10.1186/1297-9716-44-20

**Published:** 2013-03-18

**Authors:** María A Risalde, Verónica Molina, Pedro J Sánchez-Cordón, Fernando Romero-Palomo, Miriam Pedrera, Bartolomé Garfia, José C Gómez-Villamandos

**Affiliations:** 1 Department of Comparative Pathology, Veterinary Faculty, University of Córdoba-Agrifood Campus of International Excellence (ceiA3), Edificio Sanidad Animal, Campus de Rabanales, Córdoba 14014, Spain; 2 Garfia Veterinary Laboratory S.L, Calle Varsovia, parcela 53, Pol. Tecnocordoba, Córdoba 14014, Spain

## Abstract

Resistance to respiratory disease in cattle requires host defense mechanisms that protect against pathogens which have evolved sophisticated strategies to evade them, including an altered function of pulmonary macrophages (MΦs) or the induction of inflammatory responses that cause lung injury and sepsis. The aim of this study was to clarify the mechanisms responsible for vascular changes occurring in the lungs of calves infected with bovine viral diarrhea virus (BVDV) and challenged later with bovine herpesvirus type 1 (BHV-1), evaluating the role of MΦs in the development of pathological lesions in this organ. For this purpose, pulmonary lesions were compared between co-infected calves and healthy animals inoculated only with BHV-1 through immunohistochemical (MAC387, TNFα, IL-1α, iNOS, COX-2 and Factor-VIII) and ultrastructural studies. Both groups of calves presented important vascular alterations produced by fibrin microthrombi and platelet aggregations within the blood vessels. These findings were earlier and more severe in the co-infected group, indicating that the concomitance of BVDV and BHV-1 in the lungs disrupts the pulmonary homeostasis by facilitating the establishment of an inflammatory and procoagulant environment modulated by inflammatory mediators released by pulmonary MΦs. In this regard, the co-infected calves, in spite of presenting a greater number of IMΦs than single-infected group, show a significant decrease in iNOS expression coinciding with the presence of more coagulation lesions. Moreover, animals pre-inoculated with BVDV displayed an alteration in the response of pro-inflammatory cytokines (TNFα and IL-1), which play a key role in activating the immune response, as well as in the local cell-mediated response.

## Introduction

The bovine respiratory disease complex (BRDC) is an important problem for the cattle industry, often resulting in severe economic losses [1,2]. This fatal bovine respiratory infection is a multi-factorial disease associated with a primary viral infection followed by a secondary bacterial infection. It is frequently characterized by concurrent infections of several pathogens. The etiologic agents related to feedlot pneumonias include bovine viral diarrhea virus 1 and 2 (BVDV-1 and BVDV-2), bovine herpesvirus type 1 (BHV-1), bovine parainfluenza-3 virus, bovine respiratory syncytial virus, bovine adenovirus, bovine coronavirus, *Mannheimia haemolytica*, *Pasteurella multocida*, *Histophilus somni and Mycoplasma spp*. [3-6]. Resistance to respiratory disease in cattle requires host defense mechanisms that protect against viral and bacterial pathogens that have also evolved sophisticated strategies to evade the host immune responses, including among others an altered pulmonary macrophages (MΦs) function or the induction of profound inflammatory responses that cause lung injury and sepsis [6].

The inflammatory process is a protective mechanism that occurs in response to trauma, infection or tissue injury [7,8]. Increased blood supply, enhanced vascular permeability and migration of immune cells occur at damaged sites. In this process, MΦs play a central role in managing different immunopathological phenomena through the secretion of inflammatory mediators such as nitric oxide (NO), prostaglandins, and the pro-inflammatory cytokines tumor necrosis factor-α (TNFα) and interleukin (IL)-1 [9-11].

NO is a potent vasodilator, acting to maintain vascular tone and function within the vessel wall, generated from L-arginine by nitric oxide synthase (NOS) enzymes. There are three main isoforms of NOS with distinct functions and patterns of expression: endothelial NOS, neuronal NOS and inducible NOS (iNOS) highly expressed in MΦs [10,12].

Prostaglandins are other important inflammatory mediators implicated in the vascular homeostasis since its low levels can prevent or reverse aggregation of platelets and induce vasodilation [9,13]. These molecules are produced from arachidonic acid metabolites by the catalysis of cyclooxygenase-2 (COX-2) [14].

Pro-inflammatory cytokines induce integrin expression and redistribution of leukocytes, which contribute to their recruitment and activation at the site of inflammation [15,16], as well as increasing vascular permeability and tissue injury [17,18]. There is evidence that inflammatory cytokines are the main orchestrators of the inflammatory cascade in BRDC, detecting high levels of TNFα and IL-1 in the airways of cattle infected with respiratory pathogens [19-22].

BVDV is a pestivirus that although it is not a primary agent in the pathogenesis of BRDC, it can suppress the host immune system and increase the risk of secondary infections, thus enhancing pulmonary colonization by other pathogens such as BHV-1 [23-25]. The mechanisms of the immunosuppressive action of BVDV are object of debate, including changes related to decreased lymphocyte proliferation [26,27], severe lymphoid depletion in lymphoid tissues [28-30], decreased chemotaxis and phagocytic activity [31], increased production of prostaglandin E2 [32,33], increased NO synthesis in response to lipopolysaccharide [34,35] and impaired production of pro-inflammatory cytokines [30,36-39].

Therefore, the aim of this study was to clarify the mechanisms responsible for ultrastructural and histopathological changes occurring in the lungs of calves pre-infected with BVDV and challenged later with BHV-1, as well as to analyze the role of MΦs in the appearance of the lesions. Thus, this work will contribute to gaining a better understanding about how this virus predisposes to secondary airborne infections.

## Materials and methods

### Experimental design

The experimental design and collection of samples have been described by Risalde et al. [40]. Briefly, twenty-four Friesian calves (8–9 months old), BVDV and BHV-1 antigen and antibody (Ab) free, were housed in the Animal Experimental Center of Cordoba University (Spain). The animals had an adjustment period of one week before the study started and were separated into three groups based on the inoculation protocol: twelve calves were inoculated intranasally with 10 mL of a suspension of non-cytopathic BVDV-1 strain 7443 with a titration of 10^5^ tissue culture infective dose 50% (TCID_50_)/mL (courtesy of the Institute für Virologie, TIHO, Hannover, Germany). Twelve days later, when the calves showed neither clinical signs nor BVDV viraemia, they were challenged with 2 mL of BHV-1.1 strain Iowa containing 10^7^ TCID_50_/mL (courtesy of the Hipra Laboratories, Girona, Spain) (BVDV/BHV-1 group). Two calves of this group, BHV-1.1-free, were used as BVDV infection controls. At the same time, ten calves were inoculated only with BHV-1.1 (BHV-1 group). Two un-infected (UI) animals were used as negative controls and received intranasally 2 mL of tissue culture fluid free of viruses. The time point of BHV-1.1 inoculation was defined as day 0.

After virus inoculation, clinical examination was performed daily. In order to determine the number of platelets, EDTA blood obtained from coccygeal vein and nasal swab samples were collected at 0, 3, 6, 9, 12, 15, 18 and 21 hours post-inoculation, 1, 2, 4, 5, 7, 9, 12 and 14 days post-inoculation (dpi) with BHV-1.1. The infected calves were sedated with xylazine (Rompun® 2% solution; Bayer Healthcare, Kiel, Germany) and euthanized by overdosing with thiopental-sodium (Thiovet®; Vet Limited, Leyland, Lancashire, UK) in batches of two at 1, 2, 4, 7 and 14 dpi. Two animals of the BVDV/BHV-1 group were killed at 0 dpi just before BHV-1.1 inoculation (BHV-1.1-UI), being used as BVDV infection controls for this group. On the contrary, the two negative control animals were euthanized at the end of the study, being used as UI controls for the BHV-1 group. The experimental procedure was carried out in accordance with the Code of Practice for Housing and Care of Animals used in Scientific Procedures, approved by the European Economic Community in 1986 (86/609/EEC amended by the directive 2003/65/EC).

All euthanized calves were subjected to necropsy examination. The samples were collected from the lungs and immediately frozen at −80°C for virological study; likewise, they were fixed in 10% buffered formalin solution for histopathological and immunohistochemical studies as well as in 2.5% glutaraldehyde in 0.1 M PBS for ultrastructural analysis.

Lung tissue samples were analyzed to discard any infection with secondary bacterial pathogens, being subjected to microbiological routine cultures (Xylose lysine deoxycholate agar, MacConkey agar and Blood agar) by using standard procedures. No bacteria were isolated from these samples.

### Polymerase chain reaction (PCR)

RNeasy Lipid tissue kit (Qiagen) was used to extract BVDV ribonucleic acid (RNA) from lung samples, according to the manufacturer’s instructions. This RNA was subjected to a one step Real-Time Reverse Transcription plus Polymerase Chain Reaction (RT-PCR) using primers and Taqman probes (at the same concentration) from conserved regions of the 5’-UTR of BVDV-1a [41]. The reaction was performed using the Real Time Ready RNA Virus Master (Roche, Mannheim, Germany) in a LightCycler 1.5, according to the manufacturer’s instructions. The samples with a cycle threshold value less than or equal to 45 were considered as positive. The positive control was the NCP BVDV-1 strain 7443 at 10^5^ TCID_50_/mL.

A real-time PCR analysis of the BHV-1 deoxyribonucleic acid (DNA), extracted from lung samples using Genomic DNA from a tissue kit (Macherey-Nagel, Germany), was performed according to the OIE Terrestrial Manual [42]. After analyzing the results on an Applied 7300 detector (Applied Biosystems, USA), any sample with a cycle threshold value less than or equal to 45 was considered as positive. The positive control was the BHV-1.1 strain Iowa at 10^8.3^ TCID_50_/mL.

### Tissue sample collection and processing for transmission electron microscopy (TEM)

Glutaraldehyde-fixed samples were post-fixed in 2% osmium tetroxide, dehydrated in acetone and embedded in Epon 812® (Fluka Chemie AG, Buchs, Switzerland). Sections (50 nm) were counterstained with uranyl acetate and lead citrate, and examined with a Philips CM-10 transmission electron microscope.

### Immunohistochemical methods

Formalin-fixed samples were dehydrated through a graded series of alcohol to xylol and embedded in paraffin wax by routine techniques for light microscopy. Samples were sectioned (3 μm) and stained by different methods as haematoxylin-eosin and the Fraser Lendrum technique, or processed for their immunohistochemical study using the avidin-biotin-peroxidase complex (ABC) method.

The ABC method for immunohistochemistry (IHC) was performed on serial sections of formalin-fixed samples, which were dewaxed and rehydrated as previously described by Pedrera et al. [29,38]. Briefly, endogenous peroxidase activity was exhausted by incubation with 0.3% hydrogen peroxide in methanol for 30 min at room temperature. The samples were subjected to different methods for antigen retrieval (Table 1). After pre-treatment, the sections were rinsed three times in PBS (pH 7.2) for 10 min and then covered with 1% normal horse serum (Pierce-Endogen, Woburn, USA) in 0.05 M Tris buffered saline (TBS) (pH 7.6) or 20% normal goat serum in PBS for 30 min at room temperature, for primary monoclonal Ab(mAb) and polyclonal Ab(pAb), respectively. After this blocking stage, sections were incubated with primary Ab at 4°C overnight. After primary incubation, the slides were washed in PBS (three times for 5 min each) and then incubated with the secondary antibodies for 30 min at room temperature. Biotinylated horse anti-mouse IgG secondary Ab (Pierce-Endogen) diluted 1:200 in Tris buffer containing normal horse serum 1% was used for the primary mAb. Biotinylated goat anti-rabbit IgG secondary Ab (Vector Laboratories, Burlingame, CA, USA) diluted 1:200 in PBS containing normal goat serum 1.5% was used for the primary pAb. After three further 5 min washes in TBS, samples were incubated with the ABC complex (Vectastain® ABC Elite Kit, Vector Laboratories, CA, USA) for 1 h at room temperature. All tissue sections were finally rinsed in PBS and incubated with chromogen solution (NovaRED® Substrate Kit, Vector Laboratories). Slides were counterstained with Mayer’s haematoxylin. Details of the primary mAb and pAb are summarized in Table 1.

**Table 1:** Detailed list of primary antibodies used in the immunohistochemical study

***Specificity***	***Antigen or cell detected***	***mAb/pAb***	***Dilution***	***Pre-treatment***	***Source***
Anti-human MΦs	Monocytes and MΦs	mAb	1:100	Protease^a^	Serotec
		(clone MAC387)			
Anti-bovine TNFα	TNFα	pAb	1: 25	TC-microwave^b^	Serotec
Anti-human IL-1α	IL-1α	pAb	1:100	Tween-20^c^	Endogen
Anti-murine iNOS/NOS Type II	iNOS/NOS Type II	pAb	1:100	TC-autoclave^d^	BD Transduction Lab.
Anti-murine COX-2	COX-2	pAb	1:75	TC-autoclave^d^	Cayman Chemical
Anti-Human Von Willebrand Factor	Von Willebrand Factor	pAb	1:800	Protease^a^	DakoCytomation

MΦs, macrophages; mAb, monoclonal antibody; pAb, polyclonal antibody.

^a^ Incubation with 0.1% protease type XIV (Sigma–Aldrich Chemie) in 0.01 M PBS, pH 7.2, for 10 min at room temperature.

^b^ Incubation with 0.1 M citric acid (pH 6), microwave for 5 min at sub-boiling temperature.

^c^ Incubation with 0.1% Tween 20 (Merck) in 0.01 M PBS, pH 7.2, for 10 min at room temperature.

^d^ Incubation with 0.1 M citric acid (pH 6) and autoclaved for 30 min at 121°C, 1 atm.

Tissue samples from cattle, in which reactivity for primary Ab against cytokines and cellular markers used in this study had been demonstrated, were used as positive controls in IHC [29,38]. Tissue sections for which the specific primary Ab were replaced by rabbit or mouse non-immune sera (DakoCytomation, Glostrup, Denmark) were used as negative controls.

### Cell counting and statistical analysis

In order to evaluate the number of immunolabeled cells and to correlate the results obtained using different Ab, two paraffin-wax blocks from the lungs of each animal were selected. Cell counts were carried out on 50 randomly chosen fields of 0.2 mm^2^ from tissue sections from these blocks. The results are given as the number of positive cells per 0.2 mm^2^. Identification of different types of immunolabeled cells was based on morphological features, location and size of the cells.

The different pulmonary MΦs exhibited rounded or elongated morphology, an indented nucleus and abundant cytoplasm. Interstitial MΦs (IMΦs), pulmonary intravascular MΦs (PIM) and pulmonary alveolar MΦs (PAM) are localized in distinct anatomical compartments of the lung, including connective tissue, being adhered to the endothelium in the pulmonary capillaries and air spaces, respectively. PIM and IMΦs were grouped together and are described as “septal MΦs”.

A semiquantitative estimation of platelet aggregation Factor-VIII-positive was performed on 50 randomly chosen 0.2 mm^2^ areas. The results are given as the presence of positive clusters of platelets per area as follows: absent (−), mild (+), moderate (++) and abundant (+++). For graphical representation and statistical analysis, they were scored from absent to severe (0 to 3).

Quantifications were performed by two experienced observers but with no previous knowledge of which group was being analyzed. Data were assessed to calculate mean ± standard error values and were analyzed with the SAS System for Windows, version 9.1 (SAS Institute, Cary, North Carolina, USA). Chi-square analysis was employed for the estimation of platelet aggregation Factor-VIII-positive for which a *p* < 0.05 was accepted as statistically significant, using a Fisher’s exact test in the instance of an expected value below 5. The Duncan’s Multiple Range Test (*p* < 0.05) was used to analyze significant differences of the values in the same group at various time points (*) and non-paired Student’s *t*-test (*p* < 0.05) was used between both inoculated groups at the same time point (**).

## Results

### Respiratory signs and hematological findings

The main respiratory signs are given in Risalde et al. [40]. Briefly, the calves pre-infected with BVDV show more intense respiratory signs such as cough, mucopurulent nasal discharge, dyspnoea and open-mouth breathing mainly between 4 and 11 dpi, while the calves of the BHV-1 group only presented a moderate serous nasal discharge.

Platelet numbers were within the clinically normal range in all calves throughout the study, except for 2 animals from both inoculated groups, where an important decrease of platelet numbers to approximately 57% was observed at 12 hpi.

### Detection of BVDV and BHV-1 in the lungs

The presence of BVDV in the lungs of co-infected calves was detected by RT-PCR between 0 and 7 dpi (12–19 dpi BVDV), presenting the samples cycle threshold values between 33 and 39; whereas BHV-1 was detected by PCR from 2 to 14 dpi in these calves with cycle threshold values from 35 to 40. On the contrary, BHV-1 was only detected between 4 and 7 dpi in the BHV-1 group, showing the samples higher cycle threshold values (between 41 and 42), while BVDV was not detected in these calves throughout the study.

### Respiratory lesions

There were no remarkable lesions in the negative control animals. The pulmonary parenchyma of inoculated calves was affected by interstitial pneumonia with alveolar septal thickening produced by interstitial aggregates of mononuclear cells. The appearance of this alteration was earlier in the co-infected calves and was associated with occasional haemorrhages.

**Figure 1:**
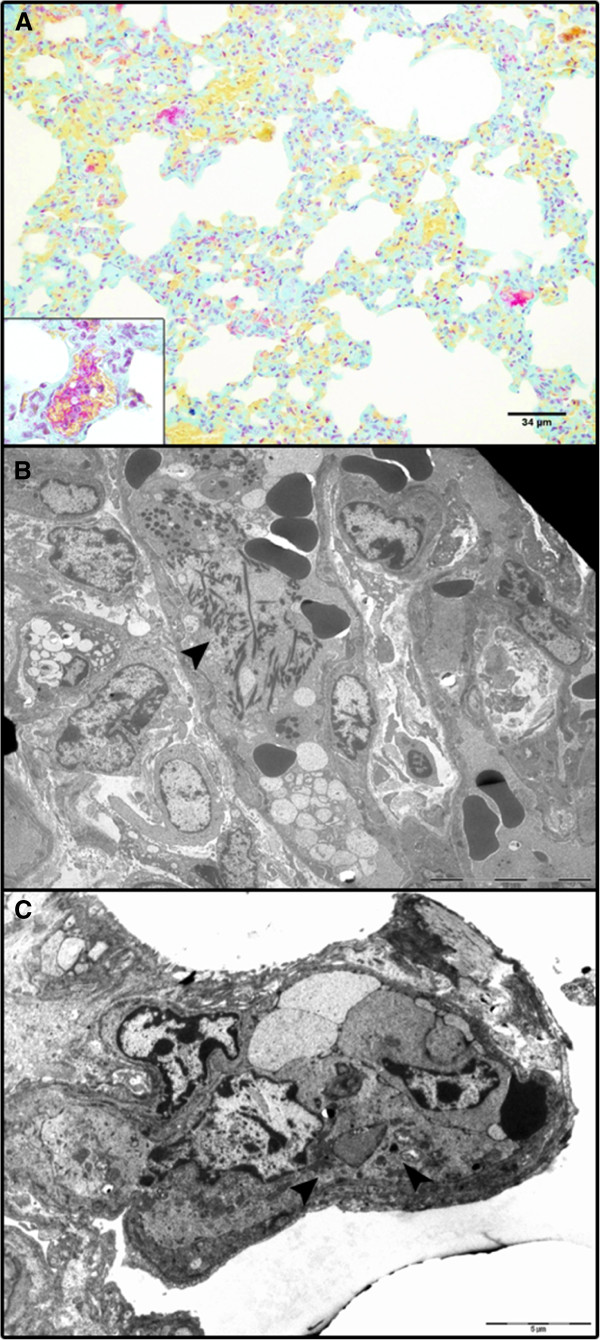
**Fibrin microthrombi in pulmonary venules of animals pre-infected with BVDV and challenged later with BHV-1.1.** Co-infected animals presented vascular alterations that facilitate the occlusion of the pulmonary venules as fibrin microthrombi observed with Fraser Lendrum technique (**A**) and TEM (**B**) at 4 dpi, as well as PIM enlarged and rounded engulfing cell debris (arrowheads) observed with TEM at 4 dpi (**C**).

The fraser Lendrum technique revealed fibrin microthrombi in some pulmonary venules and capillaries of inoculated calves, being more severe in co-infected calves, mainly from 4 dpi onwards (Figure 1A). These changes were confirmed by the ultrastructural study together with the presence of fibrin in alveoli associated with PAM in both inoculated groups at early stages (Figure 1B). Moreover, these animals displayed an intense hyperaemia as well as interstitial and alveolar oedema during this period.

**Figure 2:**
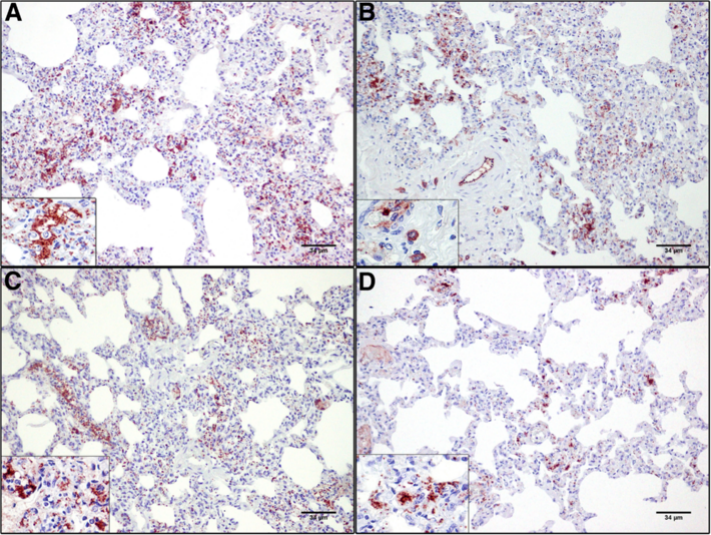
**Immunohistochemical detection of pulmonary platelet clusters in calves with and without pre-existing BVDV challenged with BHV-1.1.** Immunohistochemical study of the lung in these animals revealed that the calves of the BVDV/BHV-1 group showed a great quantity of Factor VIII-positive clusters of platelets in some pulmonary venules and capillaries at 4 dpi (**A**), compared with minor changes present in the BHV-1 group (**B**), where IMΦs and periarterial MΦs were observed engulfing positive granular material. Moreover, the calves of the BVDV/BHV-1 group showed lower quantities of platelet clusters at 14 dpi (**C**), with this lesion in the BHV-1 group being almost inexistent at this time (**D**).

**Figure 3:**
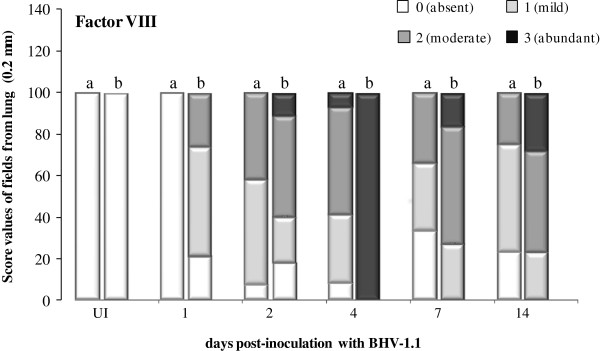
**Representative graphic of the valuation of factor VIII-positive platelet clusters detected by immunohistochemical method.** These score values were obtained for the analysis of 100 fields (0.2 mm^2^ each) from the lung of calves (*n* = 2 per time point) inoculated with BHV-1.1 (**a**) versus calves inoculated with BVDV and BHV-1.1 (**b**). The results are given as follows: absent (−), mild (+), moderate (++) and abundant (+++), and scored from absent to severe (0 to 3). (UI, BHV-1.1 un-infected: negative controls for the BHV-1 group and BVDV infection controls for the BVDV/BHV-1 group).

These findings were associated with a great quantity of platelets, whose detection was performed by IHC using anti-Factor-VIII Ab. Thus, in the negative control calves, this Ab prompted positive granular immunostaining among sheathed capillary cells, free in the interstitium and occasionally in MΦs cytoplasm. However, in the inoculated groups, clusters of immunostained granular material were observed in blood vessels. Moreover, numerous IMΦs and periarterial MΦs, together with some PAM, were swollen and displayed an intense positive granular and cytoplasmic reaction in both inoculated groups between 4 and 7 dpi (Figure 2A, 2B). In the BVDV/BHV-1 group, clusters of platelets were observed from 1 dpi, being more evident throughout the study associated with MΦs engulfing platelets that peaked at 4 dpi (score value of 3 in all evaluated fields) (Figure 3). Subsequently, the positive reaction in MΦs and vascular lumina diminished considerably without recovering thereafter (Figure 2C). There was a significant difference in the presence of platelet clusters in this group throughout the study (*p* = 0.0119). In the BHV-1 group, these findings were observed from 2 dpi with similar values to the co-infected group, decreasing the number of platelet clusters until the end of the study (Figures 2D and 3). However, there was no significant difference in the incidence of these clusters in the lungs of animals infected only with BHV-1.

**Figure 4:**
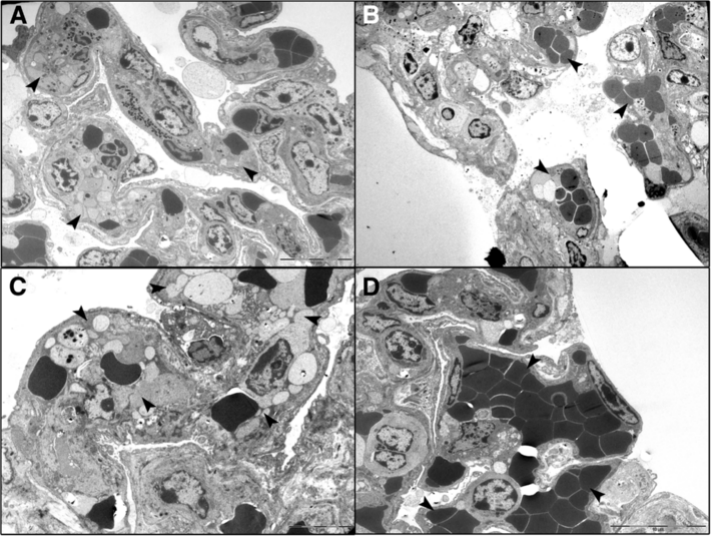
**Ultrastructural detection of pulmonary platelet clusters in calves with and without pre-existing BVDV challenged with BHV-1.1.** The analysis of the samples with transmission electron microscopy confirmed the presence of multiple aggregations of platelets within the blood vessels with some vascular lumina completely occluded (arrowheads) in the lungs of the BVDV/BHV-1 group at 4 dpi (**A**), versus a mild lesion (arrowheads) observed in the BHV-1 group (**B**). Pulmonary parenchyma showed recovery signs of this lesion (arrowheads) in the co-infected group at 14 dpi (**C**), in comparison with a total recuperation (arrowheads) in the single infection (**D**).

Ultrastructural studies confirmed that there was an increase in the number of activated platelets forming multiple aggregations within the blood vessels, whose vascular lumina usually appeared completely occluded. The changes indicative of this activation were an enlarged and deformed shape of the cells, a partial or total decrease in granule numbers and a dilation of the open canalicular system. The most common form of platelet aggregation was the appearance of mosaic-like clusters comprising fully degranulated platelets with fusion of cytoplasmic membranes. Occasionally, these membranes completely disappeared, giving rise to a finely granular structure with low electron density, containing vestiges of platelet organelles and surrounded by a membrane layer. In the BVDV/BHV-1 group, platelet aggregation occurred sooner and was more intense, increasing between 4 and 7 dpi (Figure 4A) and not showing a total recovery at the end of the study (Figure 4C). In the BHV-1 group this lesion was moderate, presenting retrieval signs from 7 dpi (Figure 4B, 4D).

Subcellular changes indicative of a slight secretory activation in IMΦs such as enlargement, proliferation and dilation of rough endoplasmic reticulum and Golgi complex cisternae were mainly observed in the BHV-1 group at 4 dpi. Moreover, from 4 dpi, some PIM and PAM of both inoculated groups appeared enlarged and rounded, with loss of filopodia, increased number of lysosomes and varying amounts of cell debris in their cytoplasm, characteristic signs of phagocytic activation (Figure 1C).

**Figure 5:**
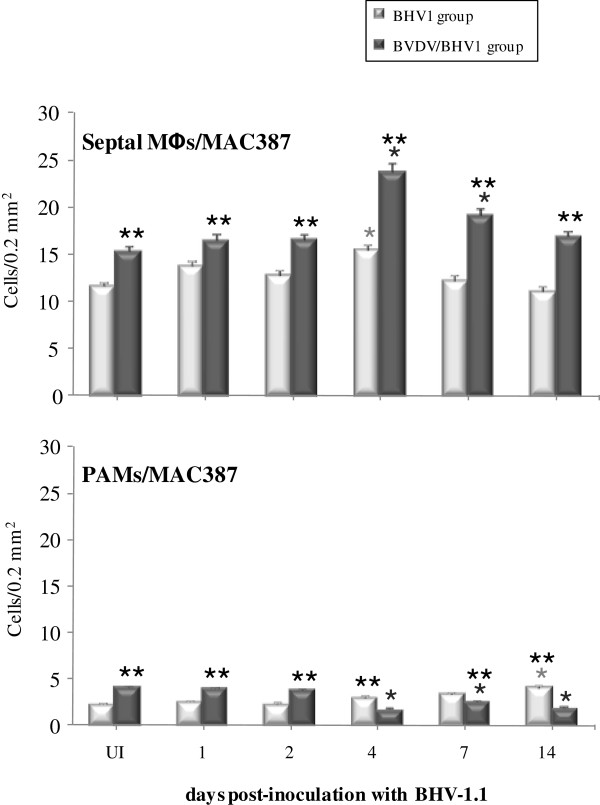
**Septal MΦs and PAM (means ± standard errors) positive for MAC387 by immunohistochemical method.** The values were evaluated in the lung of calves co-infected experimentally with BVDV and BHV-1.1 compared with calves inoculated only with BHV-1.1 (*n* = 2 per time point). (UI, BHV-1.1 un-infected: negative controls for the BHV-1 group and BVDV infection controls for the BVDV/BHV-1 group. *p < 0.05 significant differences in the same group at various time points; **p < 0.05 significant differences between inoculated groups at the same time point).

Immunolabeling of septal MΦs displayed similar kinetics in single and dual infections after BHV-1.1 inoculation, although presenting differences in the magnitude of their response. Thus, the BVDV/BHV-1 group showed a higher number of these cells during the study, peaking at 4 dpi (*p* < 0.0001). The number of PAM was also significantly higher in the co-infected calves at the start of the study, showing a decrease from 4 dpi (Figure 5).

Secretory activity of MΦs observed ultrastructurally was confirmed by IHC, which enabled the detection of MΦs-secreted inflammatory mediators.

**Figure 6:**
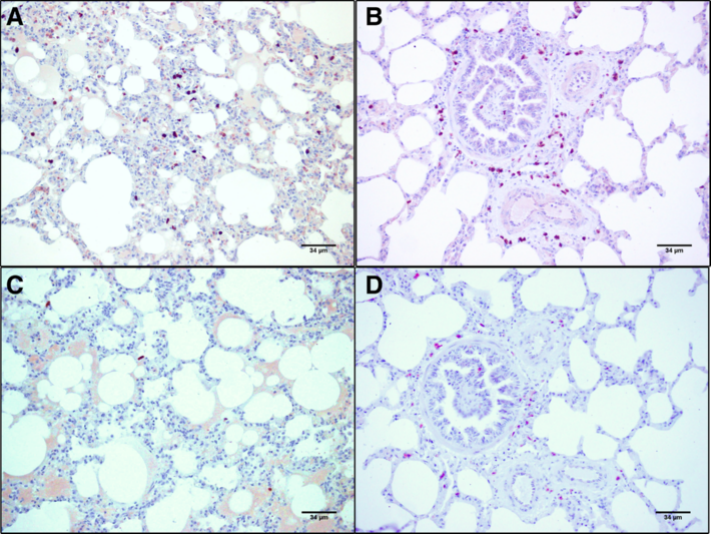
**Pro-inflammatory cytokines in the lungs of calves with and without pre-existing BVDV challenged with BHV-1.1.** An immunohistochemical study revealed the presence of septal MΦs and PAM positive for TNFα (**A**) and IL-1α (**C**) associated with sites of inflammation in the pulmonary parenchyma of the BVDV/BHV-1 group at 2 dpi. However, the calves of the BHV-1 group presented a higher number of IMΦs reactive to TNFα (**B**) and IL-1α (**D**) in the peribronchial areas of the lung at 2 dpi.

**Figure 7:**
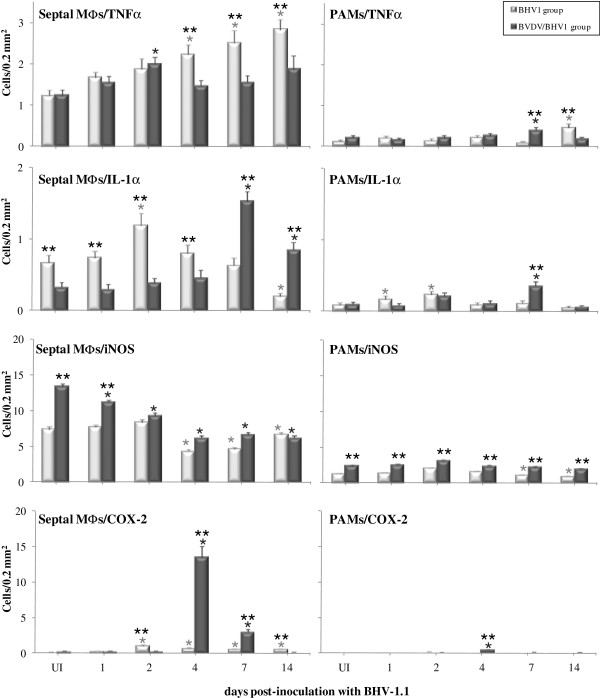
**Septal MΦs and PAM (mean ± standard error) positive for TNFα, IL-1α, iNOS and COX-2.** Immunohistochemical study revealed changes in the lungs of calves co-infected experimentally with BVDV and BHV-1.1 compared with calves inoculated only with BHV-1.1 (*n* = 2 per time point). (UI, BHV-1.1 un-infected: negative controls for the BHV-1 group and BVDV infection controls for the BVDV/BHV-1 group. *p < 0.05 significant differences in the same group at various time points; **p < 0.05 significant differences between inoculated groups at the same time point).

TNFα and IL-1α-producing cells, identified as septal MΦs and PAM, were detected immunohistochemically in the lungs of control and infected animals. These proinflammatory cytokines presented differences in magnitude and kinetics between single and dual infections. TNFα-positive septal MΦs were associated with sites of inflammation in the BVDV/BHV-1 group (Figure 6A), showing only a slight peak at 2 dpi (*p* < 0.008), whereas the BHV-1 group presented a longer response of this chemical mediator in peribronchial areas (from 4 dpi; *p* < 0.0001) (Figure 6B). On the contrary, IL-1α-reactive septal MΦs were significantly different between both infected groups before BHV-1 inoculation (0 dpi BHV-1). BVDV/BHV-1 group calves maintained lower numbers of IL-1α-positive septal MΦs throughout the study (Figure 6C), showing a delayed response to BHV-1 inoculation (from 7 dpi onwards). By contrast, BHV-1 group calves displayed an early increase of this cytokine associated with peribronchial areas (at 2 dpi; *p* < 0.007) (Figure 6D). The number of PAM positive for studied cytokines was low in both inoculated groups, presenting only a slight response at the end of the study, with the exception of an IL-1α peak in the single infected group between 1 and 2 dpi (Figure 7).

**Figure 8:**
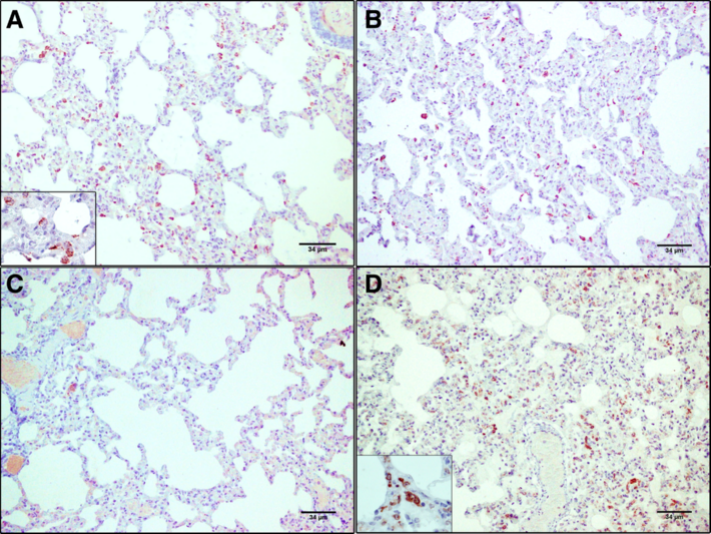
**Immunohistochemical detection of iNOS and COX-2 in the lungs of BVDV pre-infected calves challenged with BHV-1.1.** Numerous immunolabeled septal MΦs iNOS-positive were observed in the lungs of calves inoculated only with BVDV (0 dpi of BVDV/BHV-1 group) (**A**), versus a minor number of these cells at 4 dpi BHV-1.1 (**B**). Pulmonary parenchyma of calves inoculated only with BVDV (0 dpi of BVDV/BHV-1 group) showed an absence of MΦs positive for COX-2 Ab (**C**), compared with numerous immunolabeled MΦs COX-2-positive observed in the sites of inflammation at 4 dpi (**D**).

Numerous groups of immunolabeled septal MΦs presenting iNOS-positive cytoplasmic granules, mainly IMΦs, were observed in the pulmonary parenchyma of animals infected only with BVDV (Figure 8A). After BHV-1 inoculation, in BVDV/BHV-1 group calves, septal MΦs expressing iNOS decreased significantly until the end of the experiment (*p* < 0.0001), showing values of approximately twice below the baseline level from 4 dpi (Figure 8B). In healthy calves, septal MΦs presented a similar response after BHV-1 inoculation; although in these animals the decrease of this mediator was observed later than in the BVDV/BHV-1 group. On the contrary, the number of iNOS-positive PAM was higher in the BVDV/BHV-1 group and did not suffer changes throughout the study, while BHV-1 group calves showed a significant decrease from 7 dpi (Figure 7).

Intracellular localization of COX-2 was perinuclear and cytoplasmic in the MΦs, being its expression scarce in the lungs during the study (Figure 8C), except for septal MΦs of the BVDV/BHV-1 group at 4 dpi (*p* < 0.0001) (Figure 7). This intense expression occurred at sites of inflammation and injury, mainly oedemas, and was correlated with the degree of pulmonary inflammation (Figure 8D).

## Discussion

The objective of this study was to evaluate and characterize the vascular changes observed in the lungs of healthy calves and calves with subclinical BVD both experimentally inoculated with BHV-1.1, and to clarify the role of the pulmonary MΦs in the local response to the secondary pathogen.

The results show that following BHV-1 inoculation, both groups of calves displayed a mononuclear cell infiltrate and prompted major vascular alterations in the lungs, marked by intense intravascular coagulation in small and medium-sized blood vessels from an early stage of the disease. Three mechanisms, jointly known as Virchow’s triad, can induce thrombosis [43]: 1) endothelial wall injury; 2) abnormalities of blood constituents (platelets, coagulation and fibrinolytic pathways); and 3) abnormalities of blood flow.

With regard to endothelial damage, infection by human herpes simplex virus – belonging to the same subfamily that BHV-1 [44] – has been shown to cause endothelial injury, favoring the exposure of subendothelial tissue and the release of procoagulant mediators [45,46]. Here, however, neither histopathological nor ultrastructural examination disclosed any morphological evidence of endothelial damage in either of the inoculated groups.

Analysis of the cell components involved in coagulation pathways revealed fibrin deposits and intense platelet aggregation in the pulmonary microvasculature of both groups; these findings were particularly marked in the BVDV/BHV-1 group at 4 dpi, coinciding with a significant increase of the rectal temperature and severe clinical respiratory symptoms [40]. According to this, in the course of certain acute viral infections, platelets may be activated in vivo, leading to their degranulation, aggregation and withdrawal from circulation [47-49]. The procoagulant activity of BVDV and BHV-1 has been reported in vitro [50]. In our experimental study, it was increased in the co-infected calves due to the concomitance of both agents in the lungs between 2 and 7 dpi. Despite the absence of BVDV in the blood of these calves at the moment of BHV-1 inoculation, it does not completely disappear, being detected by PCR in the lungs and by IHC in lymphoid tissues throughout the experience [51]. However, there was no evidence in any stage of direct interaction between these viruses and platelets, which would suggest that platelet activation may be enhanced by indirect mechanisms including the expression of inflammatory mediators released by MΦs, which are known to play a major role in the maintenance of tissue homeostasis [11,52].

The study of pro-inflammatory cytokines revealed alterations in the kinetics and magnitude of TNFα and IL-1 expression; both mediators can prompt changes in coagulation by increasing the number of endothelial adhesion molecules or increasing vascular permeability [11,53]. Thus, the BVH-1 group calves display an increase in IL-1 synthesis by septal MΦs coinciding with the onset of platelet aggregation in the lungs (2 dpi). This, together with the subsequent action of TNFα, would favor the maintenance of the procoagulant setting. By contrast, BVDV/BHV-1 group calves, whilst exhibiting a higher number of IMΦs – the main producers of these cytokines – displayed inhibited IL-1 expression until 7 dpi, along with a minimal TNFα response. This impaired cytokine production, also observed at the systemic level [40], indicates that the synergic action of both chemical mediators can be ruled out as a potential mechanism for inducing platelet aggregation in the co-infected group.

Calves inoculated only with NCP BVDV (0 dpi for the BVDV/BHV-1 group) displayed greater expression of iNOS by septal MΦs than healthy calves [34,35,54]. However, following BHV-1 inoculation, BVDV/BHV-1 group calves exhibited an early decline in iNOS (1 dpi), an inflammatory mediator that limits the extent and duration of pathogen-induced platelet activation [55]. This finding, together with the moderate response of TNFα, may have favored the appearance of platelet aggregates in the early stages of the disease, and intense aggregation coinciding with the greatest decrease in iNOS levels (4 dpi).

The intense platelet aggregation observed in the lung microvasculature of the BVDV/BHV-1 group at 4 dpi, together with the increase in number and size of PIM as a result of phagocytic and secretory activation would indirectly prompt a slowdown in blood flow and a subsequent response by the COX-2 enzyme aimed at reversing that process [13]. However, in view of the damage observed at later stages, this action was presumably unable to counter the procoagulant events associated with the drop in iNOS expression, these being additionally enhanced by the delayed action of IL-1 in co-infected animals. Slowed blood flow, together with cytokine release, may lead to increased vascular permeability and extravasation of leukocytes into the pulmonary parenchyma [11,53,56,57].

Therefore, the results of this study indicate that the concomitance of BVDV and BHV-1 in the lungs enhances a synergic action of their pathogenic mechanisms, disrupting the maintenance of pulmonary homeostasis by facilitating the establishment of an inflammatory and procoagulant environment, characteristic of the BRDC, which appears to be modulated by inflammatory mediators released by pulmonary MΦs. In this respect, further research is required into the possible involvement of this concomitance, through the use of live BVDV and BHV-1 vaccines, in the triggering of an impaired pulmonary immune response. On the contrary, animals pre-inoculated with BVDV - despite suffering a transient infection - exhibit an alteration in the response of pro-inflammatory cytokines which play a key role in activating the immune response.

## Abbreviations

Ab: Antibody; ABC: Avidin-biotin-peroxidase complex method; BHV-1: Bovine herpesvirus-1; BHV-1 group: Calves inoculated only with BHV-1; BRDC: Bovine respiratory disease complex; BVDV: Bovine viral diarrhea virus; BVDV/BHV-1 group: Calves inoculated with BVDV and BHV-1; COX-2: Cyclooxygenase-2; DNA: Deoxyribonucleic acid; dpi: Days post-inoculation; IHC: Immunohistochemistry; IL: Interleukin; IMΦs: Interstitial macrophages; iNOS: Inducible nitric oxide synthase; mAb: Monoclonal Ab; MΦs: Macrophages; NO: Nitric oxide; NOS: Nitric oxide synthase; pAb: Polyclonal antibodies; PAM: Pulmonary alveolar MΦs; PCR: Polymerase chain reaction; PIM: Pulmonary intravascular MΦs; RNA: Ribonucleic acid; RT-PCR: Reverse transcription-polymerase chain reaction; TBS: Tris buffered saline; TCID50: Tissue culture infective dose 50%; TEM: Transmission electron microscopy; TNFα: Tumor necrosis factor-α; UI: BHV-1.1 un-infected animals.

## Competing interests

The authors declare that they have no competing interests.

## Authors’ contributions

MAR participated in the design and coordination of the experiment, performed post-mortem and IHC examinations, analyzed the data and drafted the manuscript. VM participated in the design of the experiment, carried out tissue processing and participated in the IHC examinations and data analysis. PJSC participated in the design and coordination of the experiment, coordinated tissue sample collection at post-mortem and tissue sampling. FR carried out clinical monitoring and post-mortem examinations and helped with tissue sampling. MP carried out the clinical monitoring and tissue sampling at post-mortem. BG performed the molecular techniques. JCG conceived and coordinated the study, carried out the ultrastructural study and coordinated the drafting of the manuscript. All authors read, commented on manuscript drafts and approved its final version.
